# Repurposing 6‑Anilinopurine Derivatives That Exhibit PfHDAC1 Inhibition and Antimalarial Activity against Asexual and Sexual Stages of *Plasmodium falciparum*

**DOI:** 10.1021/acsomega.5c12744

**Published:** 2026-07-06

**Authors:** Bárbara K.M. Dias, Pedro N. Maiolini, Natacha Diesca Santos, Karoline B. Waitman, Maurício T. Tavares, João P.F. Verotti, Mônica F.Z.J. Toledo, Thales Kronenberger, Roberto Parise-Filho, Célia R.S. Garcia

**Affiliations:** † Department of Clinical and Toxicological Analyses, School of Pharmaceutical Sciences, University of São Paulo, São Paulo 05508-000, Brazil; ‡ Department of Pharmacy, School of Pharmaceutical Sciences, University of São Paulo, São Paulo 05508-000, Brazil; § Department of Cancer Biology, Dana-Farber Cancer Institute, Boston 02215, United States; ∥ Interfaculty Institute of Microbiology and Infection Medicine (IMIT), University of Tübingen, Tübingen 72076, Germany; ⊥ Partner-site Tübingen, German Center for Infection Research (DZIF), Tübingen 72076, Germany; # School of Pharmacy, Faculty of Health Sciences, University of Eastern Finland, P.O. Box 1627, Kuopio FI-70211, Finland

## Abstract

Malaria is a disease caused by protozoans of the genus *Plasmodium* that claims hundreds of thousands of lives each year. The emergence of resistant strains to the currently available antimalarials urges the need to find novel compounds capable of impairing the development or transmission of the parasites. Histone deacetylase (HDAC) inhibitors are a class of compounds largely explored in antineoplastic therapy, often acting as effective and specific tumor-targeted drugs. In this work, we investigated the antimalarial activity of 6-Anilinopurine derivatives originally developed for their anticancer chemotherapeutic properties. Our results show that the compounds exhibit nanomolar activity against the asexual stages of *Plasmodium falciparum* wild-type and chloroquine-resistant strains *in vitro*. Moreover, 13 out of 14 compounds tested showed gametocidal activity after 48 h treatment, indicating their ability to inhibit the transmission of parasites from the human host to the mosquito vector. The activity of these compounds is attributed to the inhibition of *P. falciparum* HDAC1, an important enzyme in the parasite’s development. These results highlight that repurposing the use of HDAC inhibitors has significant potential in antimalarial therapy.

## Graphical abstract

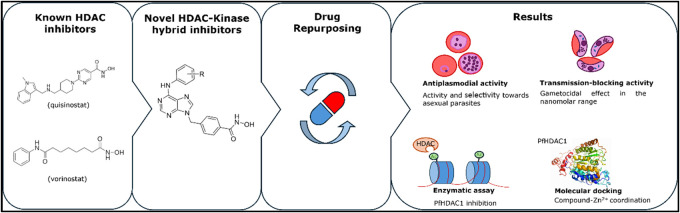

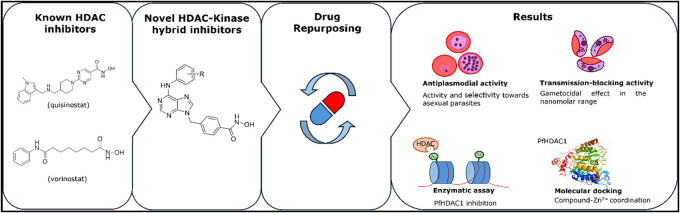

## 1 Introduction

Malaria remains a major public health problem, with millions of people at risk of infection and with over six hundred thousand deaths estimated around the world in 2024.^1^ This disease is caused by the protozoan parasite *Plasmodium*, and six species were reported to infect humans: *Plasmodium falciparum*, *Plasmodium vivax*, *Plasmodium malarie*, *Plasmodium knowlesi*, *Plasmodium ovale*, and *Plasmodium cynomolgi*.^2^ The parasite’s life cycle is complex and involves asexual replication in the vertebrate host and sexual replication in the mosquito vector. The infection starts when *Anopheles* mosquito females, during a blood meal, injects sporozoites into the vertebrate host. These sporozoites migrate and infect the hepatocytes, where they differentiate and divide, giving rise to merozoites, which, once released into the bloodstream, invade erythrocytes, initiating the asexual intraerythrocytic cycle.^3,4^ During the intraerythrocytic cycle, the parasite develops into the well-characterized stages of ring, trophozoite, and schizont,^5^ which ruptures the erythrocyte, releasing new merozoites into the blood to invade new red blood cells and continue the infection. A small proportion of the parasites commit to sexual development and, instead of further proliferating, differentiate into gametocytes. Mature gametocytes can infect the mosquito vector and circulate in the bloodstream for several days, increasing the likelihood of parasite transmission to the mosquito vector.^6,7^

The emergence and spread of parasites resistant to the available antimalarial therapies have already been reported and represent a constant challenge in the treatment and control of the disease.^8–10^ Different factors can contribute to the emergence of drug resistance in the parasite, such as drug pressure at different life-cycle stages, stage specificity of the drug, host immunity, and the essentiality of the targeted pathway in the parasite, mosquito, and vertebrate host.^8^ Research efforts to combat the disease led to the first malaria vaccine with around 30% efficacy, RTS,S/AS01, which was approved in 2021.^11^ A second vaccine, R21/Matrix-M, was approved by WHO in 2023 and showed around 75% efficacy against clinical malaria at seasonal sites and 67% efficiency in standard sites.^12,13^ The two available vaccines are important tools for combating malaria, although their use is prioritized by young children, indicating the need to develop new chemotherapeutics to fight the disease.

Some FDA-approved drugs have been investigated for antimalarial activity, demonstrating the potential of repurposing antibiotics and other chemotherapeutics as antimalarials.^11^ Recent studies showed that the FDA-approved anticancer 5-fluorouracil presented IC_50_ in the low micromolar range against *P. falciparum* 3D7 and chloroquine-resistant strain PfK1 *in vitro*, and it also showed promising activity *in vivo* against mice infected with *Plasmodium yoelii* N67.^14^ The strategy of drug repurposing has attracted attention in recent years, as it can help identify potential therapies for diseases more quickly and at lower cost.^15^

Understanding the parasite’s cellular processes is essential for the identification of potential drug targets and the development of new drugs. Throughout its life cycle, the parasite undergoes distinct stages and inhabits different microenvironments. To adapt to environmental challenges and ensure the progression of infection, the cellular processes are highly orchestrated and coordinated in malaria parasites. One key process is the regulation of gene transcription by epigenetic modifications.

Histone deacetylases (HDACs) are key players in epigenetic regulation of gene transcription, removing acetyl groups from lysine residues in histone and nonhistone proteins, leading to chromatin condensation and transcriptional repression.^16^ In *P. falciparum*, HDAC classes I, II, and III have been reported.^17^ Class I HDAC uses Zn^2+^ as a cofactor and is located in the nucleus and the cytoplasm. *Pf*HDAC1, a class I HDAC, is mainly transcribed in trophozoites, schizonts, and gametocytes^18^ and regulates developmental processes such as schizogony, gametocytogenesis, and hepatocyte invasion. In *P. falciparum* *, Pf*HDA1 and *Pf*HDA2 represent the class II HDACs, which are both nuclear and cytoplasmic, and are enriched in the Golgi apparatus where they function as regulators of antigenic variation and gametocytogenesis.^17^ Lastly, class III HDACs or sirtuins are unusual histone deacetylases that use NAD^+^ as a cofactor. In *P. falciparum*, the sirtuins regulate the maintenance of telomeric DNA and the expression of virulence genes. In addition, they also act as sensors of nutrient fluxes, connecting the environmental factors and gene transcription.^17^

The exploration of histone deacetylase (HDAC) inhibition in *P. falciparum* began with classical hydroxamate inhibitors such as vorinostat (**1**, SAHA) and trichostatin A (**2**, Figure 1A), which induced global histone hyperacetylation and parasite death, suggesting inhibition of parasite HDAC activity, although these early compounds displayed modest selectivity indices (SIs typically <30).^19^ The repositioning of anticancer HDAC inhibitors introduced pracinostat (**3**, SB939), which inhibited *P. falciparum* at ∼100–200 nM, induced histone hyperacetylation, and was active against liver stages and in murine models, confirming on-target epigenetic effects likely mediated by parasite HDACs.^20^

**Figure 1:**
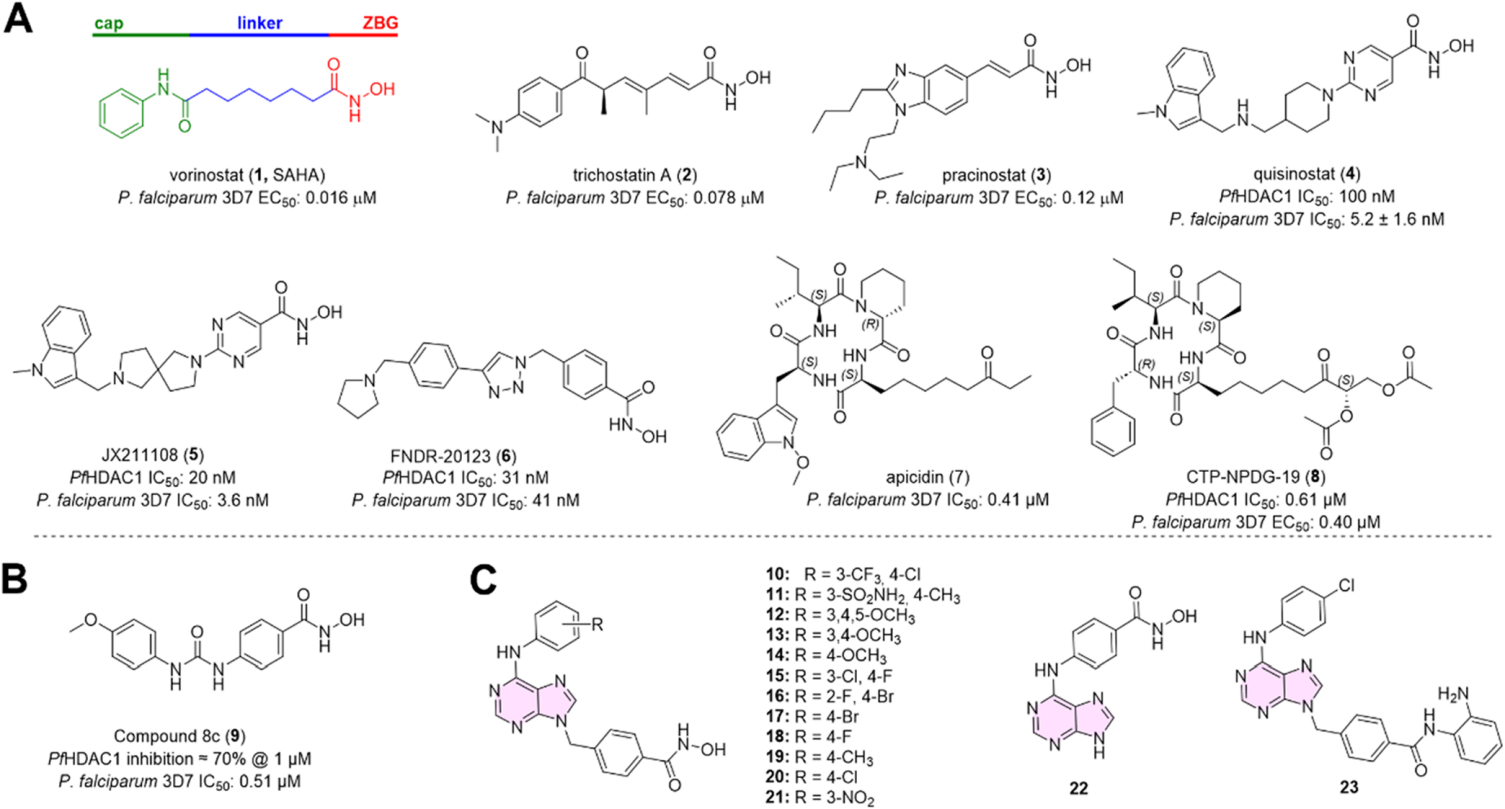
(A) Pharmacophoric group of HDAC inhibitors and representative reference scaffolds; (B) Example of 1,3-diphenylureido hydroxamates; (C) Novel 6-anilinopurine derivatives (compounds **10–23**) investigated as potential antiplasmodial agents.

A major advance came with optimization of quisinostat^4^ into JX21108 (**5**), which achieved EC_50_ values of 3.6–4.0 nM in blood stages, high selectivity (SI ∼ 1060 in HepG2 and ∼ 331 in 293T), multistage efficacy (blood, liver, and gametocytes) and strong activity *in vivo* against *P. yoelii*- infected mice with no relapse of the disease observed after treatment of 90 mg/kg, indicating that JX21108 can completely inhibit parasite in a mouse model and strongly supporting *Pf*HDAC1 as the main molecular target.^21^ Another potent *Pf*HDAC1 inhibitor, FNDR-20123, displayed nanomolar activity against both wild-type and multidrug-resistant *P. falciparum* strains (IC_50_ ∼ 41 nM in blood-stage assays). The compound also demonstrated *in vivo* efficacy in a humanized SCID mouse model, and repeated-dose toxicological studies revealed no adverse clinical signs following exposure.^22^ Although the natural product apicidin (**7**) had already demonstrated proof-of-concept as a cyclic tetrapeptide HDAC inhibitor in the 1990s,^23^ only recently has this scaffold been rationally revived, and synthetic cyclic tetrapeptide HDAC inhibitors (such as CTP-NPDG-19, **8**) now show improved parasite selectivity and differentiated killing kinetics, including rapid and irreversible killing of trophozoites.^24^ More recently, 1,3-diphenylureido hydroxamates, exemplified by compound 8c (herein 9, Figure 1B), showed ∼85% inhibition of *Pf*HDAC1, low micromolar potency in both sensitive and multidrug-resistant strains (IC_50_ ∼ 0.74–0.80 μM), and minimal inhibition of human HDAC1, achieving SI values >100 and representing a promising parasite-selective scaffold.^25^ Together with recent functional genomics studies establishing *Pf*HDAC1 as essential for asexual proliferation, erythrocyte invasion, and artemisinin stress response,^26^ these findings suggest that HDAC inhibition, potentially involving *Pf*HDAC1, represents a plausible epigenetic mechanism and a chemically tractable avenue for antimalarial development.

In this study, we investigated a series of 6-anilinopurine derivatives originally conceived for anticancer research (Figure 1C). These compounds were previously synthesized and characterized by Waitman et al. 2024 and 2025,^27,28^ where their synthetic routes and enzymatic selectivity profiles were fully described. Here, we explored the potential repurposing of this chemical class as antiplasmodial agents by evaluating their activity against asexual (3D7 and Dd2) and sexual (NF54) stages of *P. falciparum*. Complementary biochemical and molecular modeling studies were conducted to investigate whether HDAC inhibition could contribute to their activity and to provide a mechanistic rationale for their multistage antiplasmodial effects.

## 2 Results

### 2.1 6-Anilinopurine Derivatives Impair Wild-Type (3D7) and Chloroquine-Resistant (Dd2) Parasite Proliferation *In Vitro*

We investigated the antiplasmodial activity of a series of 14 6-anilinopurine derivatives originally designed for anticancer research. The main subset (compounds **10**–**21**) features a 6-anilinopurine core bearing a *para*-benzylhydroxamate moiety, with diverse substituents on the aniline ring (mono- or disubstituted in the *para* and/or *meta* positions), as shown in Figure 1B. Two additional structural variants were included: compound **22**, in which the hydroxamate linker is attached directly to the purine at position 6’; and compound **23**, a 4-chloroaniline derivative containing a benzamide moiety at N7, which also retains zinc-chelating capability. This chemical diversity allowed us to examine how variations in aniline substitution pattern and zinc-binding group (ZBG) influence the antiplasmodial potency and selectivity of the series.

A detailed structure–activity relationship (SAR) can be established among the 6-anilinopurine derivatives **10–23** (Table 1 and Figure 2), revealing that subtle variations in the electronic nature and spatial distribution of the substituents on the aniline ring markedly modulate both potency and stage selectivity. Across the series, the majority of hydroxamate analogues displayed nanomolar inhibition of *P. falciparum* 3D7 and Dd2 strains, with IC_50_ values ranging from 0.064 μM to 0.217 μM, and selectivity indices (Dd2/3D7) between 0.5 and 1.7, indicating equivalent efficacy in chloroquine-resistant parasites. The parallel evaluation in HEK293T cells confirmed mammalian cytotoxicity for most compounds (EC_50_ > 5 μM), with selectivity indices (HEK293*T*/3D7) reaching up to 23.7 for the methoxy derivative **14** (4-OCH_3_) (Supporting Information Figure S1).

**Figure 2:**
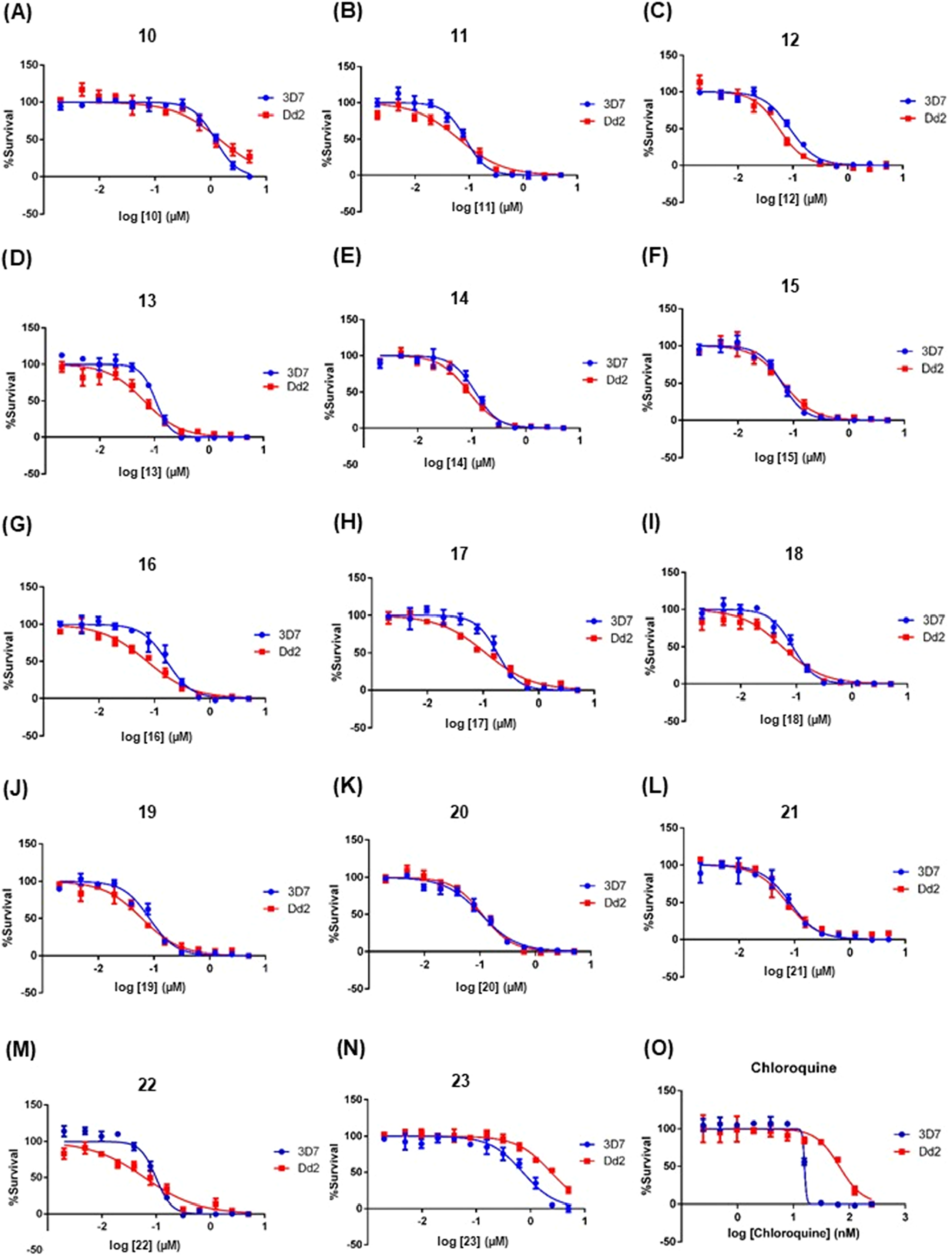
Antiplasmodial activity of compounds against *P. falciparum* 3D7 and Dd2. Dose-survival curves for compounds (A) **10**, (B) **11**, (C) **12**, (D) **13**, (E) **14**, (F) **15**, (G) **16**, (H) **17**, (I) **18**, (J) **19**, (K) **20**, (L) **21**, (M) **22**, (N) **23** against asexual blood stages of 3D7 (blue) or Dd2 (red) parasites incubated with different concentrations for 72 h, ranging from 0.05 to 5 μM. (O) Chloroquine was used as a positive control with concentrations ranging from 0.244 to 250 nM. Parasites were stained with SYBR Green I and MitoTracker Deep Red, and the final parasitemia was determined by flow cytometry. Experiments were performed three times in triplicate. Error bars represent the standard deviation.

**Table 1:** Activity of Compounds in *P. falciparum* 3D7, Dd2, HEK293T Cells, and Gametocytes IV and V Survival

				**SI** ^a^	**SI** ^b^	
**compound**	**IC**_**50**_ **3D7 (μM)**	**IC**_**50**_ **Dd2 (μM)**	****EC**_**50**_ **in HEK293T** (μM)**	**(Dd**2/3**D7)**	**(HEK293** *T* **/3D7)**	**%survival** ^c^
**10**	0.943	1.318	>5	1.397	–	48
**11**	0.086	0.060	>5	0.698	–	39.44
**12**	0.086	0.064	>5	0.744	–	47.55
**13**	0.123	0.098	>5	0.796	–	41.67
**14**	0.097	0.078	2.316	0.802	23.76	44.33
**15**	0.08	0.068	1.245	0.855	15.56	10.55
**16**	0.153	0.089	2.044	0.581	13.35	69.11
**17**	0.217	0.103	1.074	0.475	4.949	57
**18**	0.122	0.083	1.739	0.680	14.25	36.11
**19**	0.115	0.095	0.969	0.826	8.426	68.67
**20**	0.147	0.183	1.647	1.251	11.2	48.17
**21**	0.138	0.139	2.139	1.013	15.56	22.88
**22**	0.093	0.106	>5	1.140	–	68.78
**23**	1.458	1.610	3.514	1.107	2.41	96.97
**chloroquine**	0.013	0.081	–	6.192	–	–

a SI < 1 indicates slightly higher potency against the resistant strain.

b “–” = not determined. > 5 indicates no IC_50_ up to 5 μM was determined.

c Stages IV and V gametocytes were tested at saturating concentrations of 5 μM for 72 h.

Among all analogues, compound **15** (3-Cl, 4-F) exhibited the highest potency (IC_50_ = 0.080 μM in 3D7; 0.068 μM in Dd2) coupled with moderate cytotoxicity (EC_50_ = 1.24 μM; SI = 15.5) and outstanding transmission-blocking capacity, reducing mature gametocyte survival to only 10.5% at 5 μM. In contrast, the more sterically demanding dihalogenated compound **16** (2-F, 4-Br) maintained nanomolar activity in asexual parasites (IC_50_ = 0.153/0.089 μM) but lost gametocytocidal efficacy (69.1% survival), indicating that bulky substituents near the *ortho* position may hinder productive binding or parasite penetration.

The methoxy-substituted analogues **12** (3,4,5-OCH_3_), **13** (3,4-OCH_3_), and **14** (4-OCH_3_) demonstrated consistent and potent activity against both strains (IC_50_ ≈ 0.08–0.12 μM). In particular, the 4-methoxy derivative **14** combined high potency (IC_50_ = 0.097/0.078 μM) with measurable cytotoxicity (EC_50_ = 2.32 μM), affording a selectivity index of 23.8, one of the highest in the series. Nonetheless, their gametocytocidal effects were moderate (≈ 43–47% survival), suggesting that electron-donating groups may reduce accumulation or target engagement in sexual-stage parasites.

Halogenation in the *para*-position alone also maintained potency but produced distinct phenotypic outcomes. The monobrominated **17** (4-Br) and monofluorinated **18** (4-F) showed IC_50_ values of 0.217/0.103 μM and 0.122/0.083 μM, respectively, with cytotoxicity EC_50_ values of 1.07–1.74 μM (SI ≈ 5–14). Interestingly, the lighter halogen (−F) at *para*-position was associated with a more favorable gametocyte profile (36% survival) compared to −Br (57%), indicating that steric volume and polarizability of the substituent modulate transmission-stage activity. The 4-methyl analogue **19** (4-CH_3_) exhibited comparable IC_50_ (0.115/0.095 μM) but higher cytotoxicity (EC_50_ = 0.969 μM, SI = 8.4) and poor gametocidal performance (68.67% survival), confirming that nonpolar lipophilic groups alone do not translate into dual-stage efficacy.

Substituents with strong electron-withdrawing character produced divergent effects. The 3-nitro analogue **21** (3-NO_2_) retained potent antiplasmodial activity (IC_50_ = 0.138/0.139 μM), slightly mammalian selectivity (EC_50_ = 2.139 μM; SI = 15.6), and one of the best gametocidal profiles (22.9% survival). In contrast, derivative **10** (3-CF_3_, 4-Cl) was markedly less potent (IC_50_ = 0.943/1.318 μM) and offered no gain in selectivity or transmission blocking (48% survival).

Linker and ZBG modifications provided further mechanistic insights. The N6-linked hydroxamate **22** preserved strong asexual activity (IC_50_ = 0.093/0.106 μM). It was nontoxic (EC_50_ > 5 μM). Yet, its gametocytocidal effect was minimal (79.7% survival), suggesting that linker flexibility and the orientation of the ZBG are crucial for achieving or maintaining the compound in sexual stages. In contrast, replacing the hydroxamate with a benzamide ZBG, as in **23** (4-Cl, benzamide), resulted in a drastic potency loss (IC_50_ = 1.46/1.61 μM) and high gametocyte survival (96.9%), confirming that a strong Zn^2+^-chelating group is essential for antiplasmodial efficacy.

Overall, compounds **12** (3,4,5-OCH_3_) and **13** (3-Cl, 4-F) emerged as representative leads of the series. While **12** displayed the most favorable selectivity and was later confirmed as a *Pf*HDAC1 inhibitor, **15** (3-Cl, 4-F) combined strong potency with exceptional transmission-blocking activity, standing out as the phenotypic lead.

### 2.2 6-Anilinopurine Derivatives Show Gametocidal Effect against NF54 Stages IV and V *In Vitro*

To evaluate the activity of the compounds against the sexual stages of *P. falciparum*, mature stage IV–V gametocytes (NF54) were incubated with 5 μM of each compound for 72 h. All compounds except **23** (4-Cl, benzamide) impaired gametocyte survival. Among them, compounds **16** (2-F, 4-Br), **17** (4-Br), **19** (4-CH_3_), and **22** exhibited reduced activity with survival rates above 50% (Table 1 and Figure 3A). Compound **15** (3-Cl, 4-F) showed the highest activity, decreasing gametocyte survival to approximately 10.5%.

**Figure 3:**
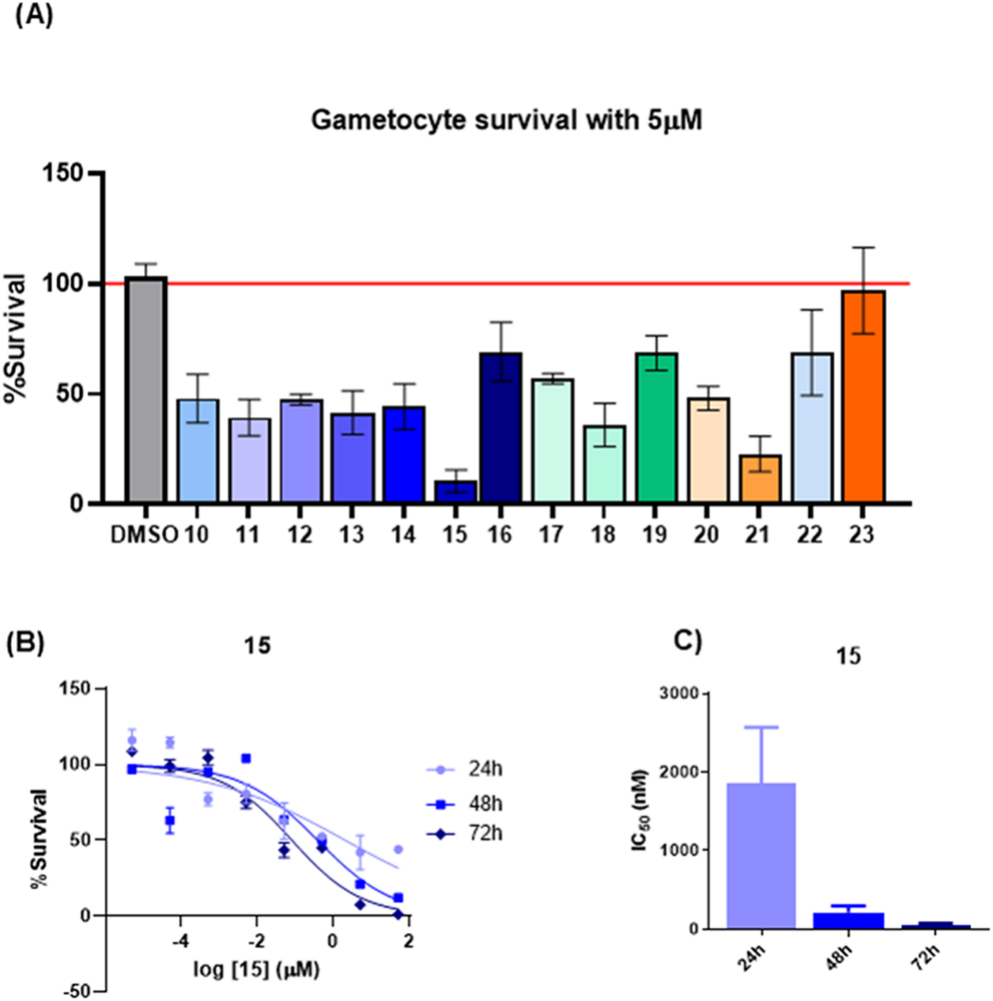
6-Anilinopurine derivatives’ effect on NF54 gametocytes. (A) Percentage of survival of NF54 stages IV and V gametocytes incubated with 5 μM of each compound for 72h. (B)­Time-dependent dose-survival curves for compound **15** (3-Cl, 4-F). (C) Time-dependent variation in IC_50_ value for compound **15** (3-Cl, 4-F).

To investigate whether **15** (3-Cl, 4-F) activity against gametocytes would be time-dependent, stages IV and V gametocytes were treated with increasing concentrations of the compound for 24, 48, or 72 h. The results obtained show that **15** (3-Cl, 4-F) activity against the sexual stages IV and V of *Plasmodium* is time-dependent (Figure 3B,C), with an IC_50_ in the micromolar range (1.84 μM) after 24 h incubation, which increases in potency toward the nanomolar range in 48 h (IC_50_: 212.4 nM) and 72 h (IC_50_: 56.68 nM).

### 2.3 6-Anilinopurine Derivatives Can Stably Bind to *Pf*HDAC1

The potential binding mode for the most potent compounds **11** (3-SO_2_NH_2_, 4-CH_3_), **12** (3,4,5-OCH_3_), **15** (3-Cl, 4-F), and **22** to *Pf*HDAC1 was studied using molecular modeling. Briefly, we previously generated a homology model of *Pf*HDAC^25^ (Figure 4A,B) and used it for docking compounds in the shallow surface pocket. The binding mode for all inhibitors was modeled as bidentate. When a hydroxamate inhibitor binds in a bidentate manner, it displaces the catalytic water and coordinates Zn^2+^ with its hydroxyl oxygen (deprotonated) and carbonyl oxygen. In addition, Zn^2+^ is coordinated in place by Asp174, His176, and Asp262. Indeed, our previous *Pf*HDAC1 simulations, in which we analyzed both mono- and bidenticity, the latter generated stable binding conformations.^25^ Those restrictions were imposed to isolate the effects arising from different cap changes. Those binding mode models underwent classical MD simulations. The predicted binding energy and interaction frequency along the trajectory were used as parameters for the binding discussion.

**Figure 4:**
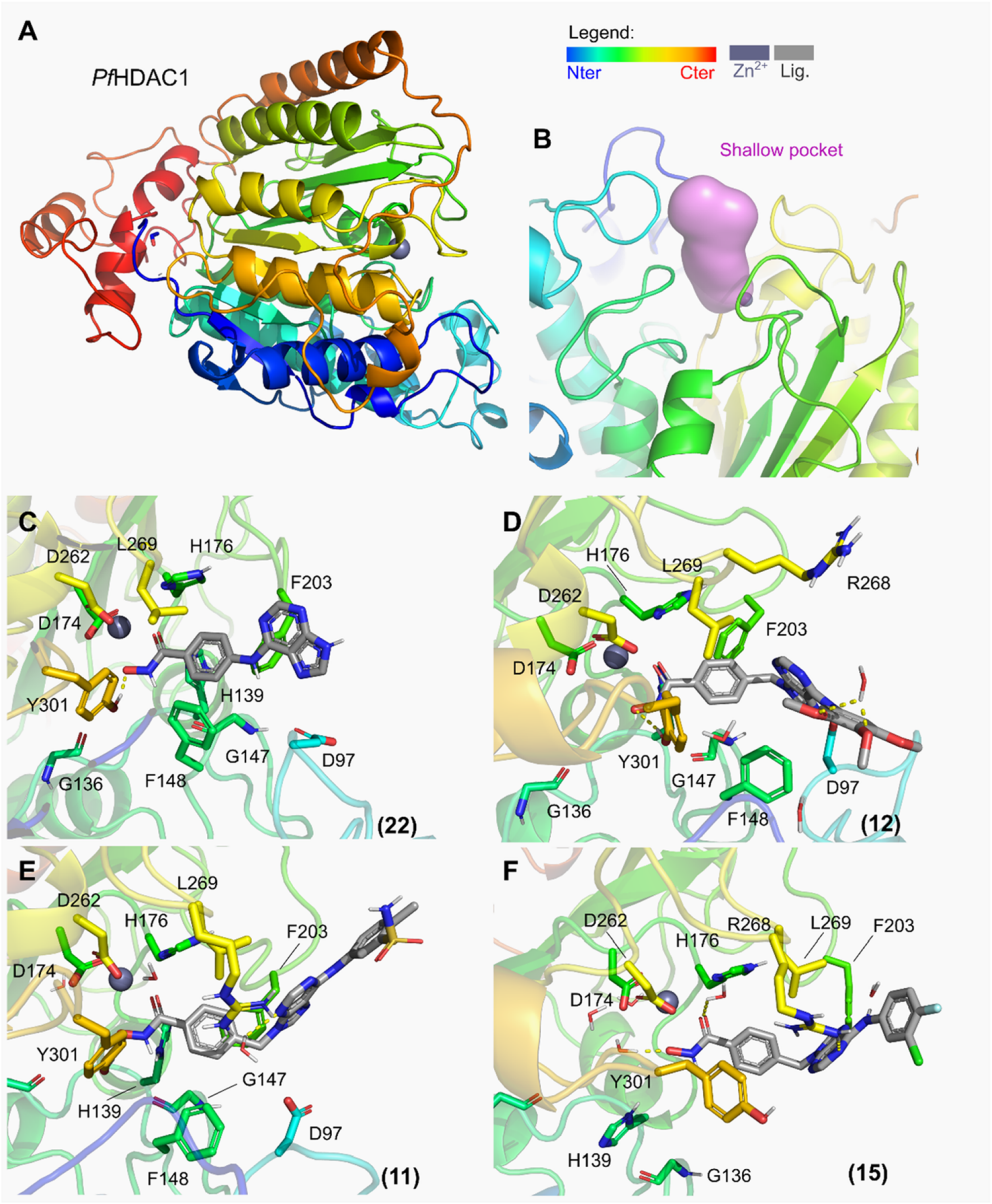
Potential binding modes of relevant 6-anilinopurine derivatives within *Pf*HDAC1. (A) Overview of the *Pf*HDAC1 model (Met1-Tyr449) colored by sequence and depicting the shallow surface binding pocket (as a pink shadow). (B). Potential binding mode for relevant HDAC inhibitors within this binding site for: **22** (C), **12** (3,4,5-OCH_3_) (D), **11** (3-SO_2_NH_2_, 4-CH_3_) (E), and **15** (3-Cl, 4-F) (F), retrieved by the most populated cluster during the simulation (see methods).

The proposed binding mode of 6-anilinopurine derivatives within *Pf*HDAC1 is similar to that previously suggested for *Hs*HDAC6,^29^ in several aspects, but not the linker. The compounds’ zinc-binding group is stabilized by interactions with Tyr301 and Glys147, while its phenyl linker establishes pi-mediated interactions with His139 and His176 (Figure 3C–F and Table S1, Supporting Information). Due to the shallow and solvent-exposed characteristic of its binding pocket, the compound’s cap mostly interacts with the solvent, being oriented by a single hydrophobic contact with Phe203. This contrasts with our previous results with human HDACs, where the cap fragments’ stability relied on hydrophobic and pi-mediated contacts. The contribution of cap interactions is highlighted on the **22** simulations, displaying the poorest predicted binding energy of the series (10.7 kcal/mol, Table S1) due to the low contribution of lipophilic terms. All other compounds have reasonably predicted binding energies ranging from high energies such as −6.3 kcal/mol (**15**) (3-Cl,4-F) toward the lowest values on **12** (3,4,5-OCH_3_, −14.7 kcal/mol, Table S1).

To validate the inhibition of PfHDAC1 by compound **12** and compound **15** as predicted, we performed an enzymatic assay, based on a fluorogenic substrate and developer, using the parasite recombinant PfHDAC1 and the human recombinant rhHDAC1 and rhHDAC8 enzymes *in vitro*. The biochemical assay confirmed direct inhibition of *Pf*HDAC1 by compounds **12** (3,4,5-OCH_3_) and **15** (3-Cl,4-F). Compound **12** inhibited the parasite enzyme activity in 99% with 10 μM and 96% with 1 μM. Moreover, inhibition of the human enzymes tested was similar to that observed against the parasite, with 99 and 96% inhibition of rhHDAC1 and 97% and 90% inhibition of rhHDAC8 with 10 and 1 μM, respectively. Compound **15** showed 98 and 97% inhibition against PfHDAC1 and rhHDAC1 with 10 μM and 1 μM, respectively. Although the inhibition of rhHDAC8 was slightly lower, with 94% inhibition with 10 μM and 73% with 1 μM. For comparison, the reference HDAC inhibitor vorinostat (**1**, SAHA) achieved 91% inhibition at 0.1 μM, confirming the assay’s reliability. These results validate *Pf*HDAC1 as a relevant molecular target for this series and are consistent with the stronger binding free energy predicted for compound **12** (3,4,5-OCH_3_) in molecular modeling. Although in the concentrations tested, it was not possible to determine the parasite-human selectivity of the compounds.

## 3 Discussion

The rise of drug-resistant malaria calls for new treatment approaches.^30^ By studying the signaling networks of *P. falciparum*, we identified relevant molecular targets. These pathways combine environmental information with transcriptional programs. Disrupting parasite development can be achieved by targeting specific components of the signaling pathways.

In this line, *P. falciparum* MORC (microrchidia) acts as a central regulator of epigenetic silencing by organizing heterochromatin domains enriched with clonally variant gene families. We have recently reported that *Pf*MORC interacts with transcriptional repressors and chromatin-modifying enzymes, notably histone deacetylase 1 (*Pf*HDAC1). We further demonstrated that knockdown of *Pf*MORC disrupts this architecture, resulting in the loss of heterochromatic markers, derepression of invasion-related and virulence genes, and global transcriptional dysregulation. These findings imply that *Pf*MORC acts as a structural scaffold coupling enzymatic histone modification (via HDACs) to higher-order chromatin remodeling.^31,32^ Targeting HDACs, therefore, represents a promising strategy for antimalarial drug discovery. Identifying and characterizing HDAC inhibitors may yield novel compounds that disrupt epigenetic regulation and compromise the viability of the parasites.

We evaluated a series of 6-anilinopurine benzohydroxamates previously designed for solid and hematological tumor cell lines and repurposed their biological activity to the malaria disease. Our data show that the compounds exhibit promising activity against *P. falciparum*, both the wild-type (3D7) and the chloroquine-resistant (Dd2) strains, with IC_50_ values in the nanomolar range. Although most of the compounds tested presented cytotoxicity against HEK293T cells *in vitro*, for compounds **22** (N6 linker ZBG), **10** (3-CF_3_, 4-Cl), **11** (3-SO_2_NH_2_, 4-CH_3_), **12** (3,4,5-OCH_3_), and **13** (3,4-OCH_3_) we could not determine an IC_50_ against mammalian HEK293T cells *in vitro* within the concentrations tested, indicating some selectivity against the parasite and suggesting their potential as new antimalarials, although more efforts would be necessary to evaluate their cytotoxicity in mammalian cells at higher concentrations.

Of note, previous studies tested compounds from this series against solid and hematological tumors and observed cytotoxicity against the solid tumor HCT-116 (colorectal carcinoma) and the hematological cell lines Jurkat and Namalwa, with IC_50_ values ranging from 20 nM to 31 μM.^27^ Interestingly, one of the most promising compounds in our study, **12** (3,4,5-OCH_3_), did not show toxicity against the solid tumor cell lines HCT-116 and MCF-7 at 50 μM. Moreover, the compound exhibited IC_50_ values of 1.5 μM in Jukart cells and 0.8 μM in Namalwa cells, approximately 10 times higher than the IC_50_ in malaria parasites (Table 1). In addition, compound **15** (3-Cl, 4-F), which was very active against gametocytes, also showed high activity against hematological tumors, with IC_50_ values of 0.02 μM and 0.03 μM for Jukart and Namalwa cells.^27^

In addition, compounds also inhibited parasites at the sexual stage, acting against NF54 gametocytes in stages IV and V (Table 1). Compound **15** (3-Cl, 4-F) rapidly inhibited NF54 gametocytes, with an IC_50(NF54)_ in the micromolar range during the first 24 h of treatment, and its potency increased with treatment time (Figure 2).

The potent antiplasmodial activity and low mammalian toxicity observed for several hybrid compounds **22** (N6 linker ZBG), **10** (3-CF_3_, 4-Cl), **11** (3-SO_2_NH_2_, 4-CH_3_), **12** (3,4,5-OCH_3_), and **13** (3,4-OCH_3_) reinforce the concept that multitarget inhibition of signaling and epigenetic pathways can yield selective and durable antimalarial candidates. Notably, their efficacy against late-stage gametocytes highlights potential transmission-blocking effects, addressing one of the major gaps in the current therapies. Together, these findings show the value of combining mechanistic insight into *P. falciparum* signaling and chromatin biology with rational drug design strategies to deliver the next generation of antimalarial agents capable of overcoming resistance and preventing disease propagation.

The analysis of the hybrid series shows that small changes in the arrangement of aromatic groups have a significant impact on HDAC inhibition and antiplasmodial activity. Compounds **12** (3,4,5-OCH_3_) and **14**, with electron-donating methoxy groups, exhibit selectivity and nanomolar inhibition, which aligns with optimal zinc binding and enhanced interaction with the HDAC catalytic pocket. On one hand, molecular modeling supported a conserved bidentate Zn^2+^ coordination mode consistent with compound **12**, later corroborated by the experimental inhibition data. Their low toxicity to mammalian cells, at the concentrations tested, suggests a specific effect on the epigenetics of parasites, possibly mediated through *Pf*HDAC1. On the other hand, compound **15** (3-Cl, 4-F), with electron-withdrawing groups, exhibits rapid activity against gametocytes, suggesting a different mechanism involving redox or signaling pathways in addition to HDAC inhibition. These findings suggest that both steric and electronic properties influence *Pf*HDAC1 binding and subsequent transcriptional repression.

Despite the potent multistage antiplasmodial activity and favorable selectivity observed for some compounds in this series, it is well-recognized that the clinical translation of histone deacetylase inhibitors has frequently been limited by suboptimal pharmacokinetic properties, including poor bioavailability, metabolic instability, and off-target toxicity. These challenges have also been highlighted in the context of antimalarial HDAC inhibitors, where promising *in vitro* activity does not always translate to adequate *in vivo* exposure or efficacy.

HDAC inhibition may have led to a blockade of cell cycle progression and not solely to antimalarial activity. The observed effect does not necessarily depend on selective inhibition of PfHDAC1 but may involve additional cellular factors within the parasite. It is well-known that HDACs interact with nuclear proteins and play an important role in regulatory processes. Histone deacetylases (HDACs) are key players in the epigenetic regulation of gene transcription. This explains the observed low IC_50_ values for HDAC inhibitors in the *P. falciparum* cell cycle. Although a detailed ADME/PK evaluation was beyond the scope of the present study, these aspects represent critical next steps for lead optimization. Importantly, the structure–activity relationships identified here, particularly the influence of aniline-ring substitutions and the zinc-binding group, provide a rational framework for future medicinal chemistry efforts aimed at improving pharmacokinetic behavior while preserving antiplasmodial potency and stage-specific activity.^33,34^

## 4 Conclusion

The 6-anilinopurine benzohydroxamates described here displayed potent and selective multistage antiplasmodial activity, effectively impairing both asexual (3D7 and Dd2) and sexual (NF54) stages of *P. falciparum*. Structure–activity relationship analyses revealed that substitutions on the aniline ring strongly modulate biological responses: electron-withdrawing groups such as 3-Cl,4-F enhanced potency and transmission-blocking effects, while electron-donating methoxy groups improved selectivity toward parasite cells. The hydroxamate zinc-binding group proved essential for efficacy, as replacement with benzamide markedly reduced activity. Within the series, compound **15** (3-Cl,4-F) emerged as the phenotypic lead, combining nanomolar potency with exceptional gametocytocidal activity; compound **14** (4-OMe) achieved the highest selectivity index, and compound **12** (3,4,5-OMe) was biochemically validated as a *Pf*HDAC1 inhibitor, confirming direct engagement with an essential epigenetic enzyme. Molecular modeling supported a conserved bidentate Zn^2+^ coordination mode consistent with the experimental inhibition data. Altogether, these findings indicate that *Pf*HDAC1 inhibition contributes to the observed multistage effects, while structural tuning at the aniline ring can optimize potency, selectivity, and stage-specific activity. The 6-anilinopurine benzohydroxamate scaffold, therefore, represents a versatile and mechanistically supported platform for the rational design of next-generation antimalarial agents targeting both proliferation and transmission.

## 5 Methods

### 5.1 Compounds

The 6-anilinopurine derivatives evaluated in this study were synthesized and fully characterized as previously reported by Waitman et al.^27,28^ The series includes both benzohydroxamate (**10–22**) and benzamide (**23**) derivatives, designed as structural analogues sharing the same 6-anilinopurine scaffold but differing in their terminal zinc-binding functionalities. Briefly, the compounds were obtained through a multistep synthetic route involving nucleophilic substitution of 6-chloropurine with anilines, followed by hydroxamate or amide coupling reactions to afford the corresponding benzohydroxamate or benzamide derivatives, respectively. All compounds displayed >95% purity as determined by HPLC, and their molecular structures were confirmed by HRMS analyses. Stock solutions (10 mM) were prepared in DMSO and diluted in the corresponding assay media immediately before use.

### 5.2 *P. falciparum* Culture

*P. falciparum* 3D7 parasites and chloroquine-resistant strain Dd2 were maintained in A+ human erythrocytes in 2% hematocrit and cultivated with RPMI-1640 media (GIBCO, Billings, USA) supplemented with 0.04% gentamicin sulfate, 0.05% hypoxanthine, and 0.5% Albumax II (Gibco). *P. falciparum* NF54 was maintained in RPMI-1640 (Gibco) medium supplemented with 10% human serum A+.

### 5.3 Compounds’ Effect on Parasite Growth *In Vitro* and Flow Cytometry Analysis

*P. falciparum* 3D7 or Dd2 infected red blood cells, with 0.3% initial parasitemia and 1% hematocrit, were incubated with different concentrations of the compounds, ranging from 0.005 to 5 μM, which were obtained with a 2-fold serial dilution, for 72 h under a mixture of gases (5% CO_2_, 5% O_2_, 90% N_2_). Chloroquine was used as a positive control, with concentrations ranging from 0.244 to 250 nM. As a negative control, the solvent Dimethyl Sulfoxide (DMSO) was used (v/v) with the highest percentage of the solvent being 0.05%. After incubation, parasites were doubly labeled, with the nucleic acid marker SYBR Green I (1X, Invitrogen) and mitochondria marker based on membrane potential MitoTracker Deep Red (50 nM, Invitrogen), and incubated for 20 min at 37 °C. Final parasitemia was obtained by flow cytometry using an Accuri C6 Flow Cytometer (Becton Dickinson). 10^4^ events were collected, and final parasitemia was determined from dot plots using fluorescence filters FL-4 vs FL-1, using the Flow Jo 8.0 software. The IC_50_ values were determined from the concentration-survival curves for each compound, obtained with the GraphPad Prism 8 software.^35^ To obtain the IC_50_ of compounds, at least three independent experiments were performed in triplicate.

### 5.4 Cytotoxic Effect of Compounds in HEK293T Cells

Human Embryonic Kidney 293T (HEK293T) cells were maintained in Dulbecco’s Minimum Essential Media (DMEM, Gibco) media supplemented with 10% fetal bovine serum, 3.7 g/L of NaHCO_3_ (Sigma), 100 U/ml penicillin/and 100 μg/mL streptomycin and were grown in 75 cm^2^ flasks in monolayers. To assess the cytotoxic effect of the compounds in HEK293T cells, 10^4^ cells were plated per well in flat-bottom 96-well plates. Cells were incubated for 24 h at 37 °C, 5% CO_2_ in 100 μL of complete DMEM to allow them to adhere to the plate. Cells were incubated with concentrations ranging from 0.05 to 5 μM of compounds in 200 μL of complete DMEM for 72 h at 37 °C, 5% CO_2._

After 72 h hours of incubation with compounds, 40 μL of MTT reagent (Sigma) (solution 5 mg/mL in PBS) was added to each well, and cells were incubated for a further 3 h at 37 °C and 5% CO_2_. Media was then removed, and 100 μL of DMSO was added to each well. Plates were agitated in a shaker for 10 min to dissolve precipitates. Absorbance was read at 570 nM in a FlexStation 3 (Molecular Devices). To determine the EC_50_ of compounds, at least three independent experiments were performed in triplicate.

### 5.5 Gametocyte Assay

*P. falciparum* NF54HT-GFP-luc parasites were maintained in A+ human erythrocytes at 2% hematocrit and cultivated with RPMI-1640 medium (GIBCO, Billings, USA) supplemented with 0.04% gentamicin sulfate, 0.05% hypoxanthine, and 10% A+ human serum. The cultures were synchronized at the ring stage using 5% sorbitol treatment.^36^

To induce gametocytogenesis, at the trophozoite stage, the cultures were adjusted to 3% hematocrit and 3% parasitemia. After 24 h, at the ring stage, the parasites were subjected to 48 h of nutrient starvation stress. Once sexual differentiation was induced, the asexual parasites were eliminated from the culture using 50 mM N-acetylglucosamine (NAG) treatment for 5 days. The NAG treatment was then suspended for 3 days before incubating the gametocytes with the compounds. The gametocytes were concentrated using VarioMACS magnetic cell sorting (Miltenyi Biotec) and plated in a 96-well round-bottom plate at 1% gametocytemia and 1% hematocrit. The gametocytes were then incubated with 5 μM of each compound for 72 h. Gametocytes’ survival was determined using the Luciferase Assay System (Promega) following the manufacturer’s instructions, and mean luminescence was registered using the Tristar 5 microplate reader (Berthold).

### 5.6 Molecular Modeling

#### 5.6.1 Homology Model and Protein Preparation

The *P. falciparum* 3D7 HDAC1 was retrieved from a representative simulation frame of our previous work.^25^ Briefly, on that work, the *P. falciparum* 3D7 HDAC1 homology model was generated from the (UniProt: Q7K6A1_PLAF7, Met1-Tyr449) using Phyre2 on intensive mode with standard options. The model was validated by checking its Ramachandran plot and overall energy levels, showing low confidence for the C-terminal after Tyr449. All protein structures were prepared using the Protein Wizard Preparation tool, with standard options, and the homology model was further refined to remove steric clashes. In parallel, we qualitatively compared it against the proposed AlphaFold3 model; however, the conserved ligand binding pocket was collapsed.

#### 5.6.2 Molecular Docking

Three-dimensional ligand structures were generated with LigPrep, using Epik to predict their protonation at pH 7.0 ± 1.0; diastereoisomer configurations were derived from the synthesis. The OPLS4 force field was employed for structure generation. Docking was performed using Glide^37^ using the Zn^2+^ ion to orient the binding pocket center, employing standard precision mode. For each ligand, up to 10 poses were generated, from which we then selected the conformation for MD based on relevant interactions. The inhibitor binding was modeled as bidentate. When a hydroxamate inhibitor binds in a bidentate manner, it displaces the catalytic water and coordinates Zn^2+^ with its hydroxyl oxygen (deprotonated) and carbonyl oxygen. In addition, the Zn^2+^ is coordinated in place by the Asp174, His176, and Asp262.

#### 5.6.3 Molecular Dynamics Simulations

MD simulations were carried out by using the Desmond engine^38^ with the OPLS4 force field.^39^ The system encompassed the protein–ligand/cofactor complex, a predefined water model (TIP3P) as a solvent, and counterions (Na^+^ or Cl^–^ adjusted to neutralize the overall system charge). The system was treated in a cubic box (10 Å^3^) with a periodic boundary condition specifying the size of the box from the box edges to any atom of the protein. Short-range Coulombic interactions were calculated using 1 fs time steps and a 9.0 Å cutoff value, whereas long-range Coulombic interactions were estimated using the Smooth Particle Mesh Ewald method.^40^ Equilibration was performed as previously described.^25^ Each HDAC + Ligand system was subjected to at least 1 μs simulations (split into five replicas of 200 ns, each) with random seeds. Representative frames of the simulations were retrieved using hierarchical clustering analyses (trj_cluster.py, implemented in Maestro 2024.3, Schrödinger LCC) according to the RMSD of ligand’s heavy atoms (1 Å as cutoff). All the trajectory and interaction data are available on the Zenodo repository (code: 10.5281/zenodo.16753147, made available upon publication). MD trajectories were visualized, and figures were generated using PyMOL v.3.1 (Schrödinger LCC, New York, NY, USA).

#### 5.6.4 MD Simulation Trajectory Analyses

Protein–ligand interactions and atomic distances were calculated using the Simulation Interaction Diagram analysis pipeline (Maestro 2024.3, Schrödinger LCC). RMSD values of the protein backbone were used to monitor simulation equilibration and protein folding changes (all raw data are available in the repository). MM/GBSA binding energy calculations. Molecular mechanics with generalized Born and surface area (MM/GBSA) predicts the binding free energy of protein–ligand complexes, and the ranking of ligands based on the free energy could be correlated to the experimental binding affinities, especially in a congeneric series. Every 20th frame from the simulations was considered for energy calculations with the thermal_mmgbsa.py script. Calculated free-binding energies were normalized by the number of heavy atoms (HAC), according to the following formula: Ligand Efficiency = (Binding Energy)/(1 + ln­(HAC)).

### 5.7 *Pf*HDAC1 Enzymatic Assay

All enzymatic assays were performed by BPS Bioscience as a service (see report as Supporting Information Table S2), using the *Pf*HDAC1 (Catalog: 50063, Lot number: 170320) at a concentration of 1800 ng/reaction and the HDAC Substrate 3 at 10 μM. Briefly, all of the compounds are dissolved in DMSO. A series of dilutions of the compounds was prepared in HDAC assay buffer containing 10% DMSO, and 5 μL of each dilution was added to a 50 μL reaction, resulting in a final DMSO concentration of 1% in all reactions. The compounds were preincubated in duplicate at RT for 30 min in a mixture containing HDAC assay buffer, 5 μg BSA, HDAC enzyme, and a test compound. After 30 min, the enzymatic reactions were initiated by adding HDAC substrate (see 2.3.1) to a final concentration of 10 μM. The enzymatic reaction was carried out for 120 min at 37 °C. After enzymatic reactions, 50 μL of 2× HDAC Developer was added to each well for the enzymes, and the plate was incubated at room temperature for an additional 15 min. Fluorescence intensity was measured at an excitation of 360 nm and an emission of 460 nm using a Tecan Infinite M1000 microplate reader. HDAC activity assays were performed in duplicates at each concentration. The fluorescent intensity data were analyzed using GraphPad Prism (version 10). In the absence of the compound, the fluorescent intensity (Ft) in each data set was set to 100% activity. In the absence of HDAC, the fluorescent intensity (Fb) in each data set was set to 0% activity. The percent activity in the presence of each compound was calculated according to the following equation: Activity % = (*F*-Fb)/(Ft-Fb), where *F* = the fluorescent intensity in the presence of the compound.

## Supplementary Material



## Notes

All molecular dynamics raw trajectories, topologies, parameter files, and raw data related to the protein–ligand interactions within the simulations are publicly available in the repository: DOI: 10.5281/zenodo.16753147 (PDF)

## Abbreviations and Symbols

MD: molecular dynamics
PDB: Protein Data Bank
WHO: World Health Organisation
WT: wild-type

## References

[1] World malaria report 2025: addressing the threat of antimalarial drug resistance; Geneva World Health Organization; 2025.

[2] Saab S. A., Cardoso-Jaime V., Kefi M., Dimopoulos G. (2025). Advances in
the dissection of Anopheles–Plasmodium interactions. PLoS Pathog..

[3] Cowman A. F., Tonkin C. J., Tham W. H., Duraisingh M. T. (2017). The Molecular
Basis of Erythrocyte Invasion by Malaria Parasites. Cell Host Microbe..

[4] Ménard R., Tavares J., Cockburn I., Markus M., Zavala F., Amino R. (2013). Looking under the skin:
the first steps in malarial infection and
immunity. Nat. Rev. Microbiol..

[5] Tilley L., Dixon M. W. A., Kirk K. (2011). The *Plasmodium falciparum*-infected red blood cell. Int. J. Biochem.
Cell Biol..

[6] Venugopal K., Hentzschel F., Valkiu̅nas G., Marti M. (2020). Plasmodium asexual
growth and sexual development in the haematopoietic niche of the host. Nat. Rev. Microbiol..

[7] Voss T. S., Brancucci N. M. (2024). Regulation
of sexual commitment in malaria parasites—a
complex affair. Curr. Opin Microbiol..

[8] Blasco B., Leroy Di., Fidock D. A. (2017). Antimalarial
drug resistance: Linking *Plasmodium falciparum* parasite biology to the clinic. Nat. Med..

[9] Wicht K. J., Mok S., Fidock D. A. (2020). Molecular Mechanisms of Drug Resistance in *Plasmodium falciparum* Malaria. Annu. Rev. Microbiol..

[10] Hanboonkunupakarn B., White N. J. (2016). The threat of antimalarial
drug resistance. Trop Dis Travel Med. Vaccines..

[11] Theodoridis L., Carvalho T. G. (2025). Antimalarial drug
resistance and drug discovery: learning
from the past to innovate the future. Int. J.
Parasitol Drugs Drug Resist..

[12] Datoo M. S., Dicko A., Tinto H. (2024). Safety
and efficacy
of malaria vaccine candidate R21/Matrix-M in African children: a multicentre,
double-blind, randomised, phase 3 trial. Lancet..

[13] Okombo J., Fidock D. A. (2025). Towards next-generation treatment options to combat *Plasmodium falciparum* malaria. Nat. Rev. Microbiol..

[14] Yadav K., Shivahare R., Shaham S. H., Joshi P., Sharma A., Tripathi R. (2021). Repurposing
of existing therapeutics to combat drug-resistant
malaria. Biomedicine & Pharmacotherapy..

[15] Baker N. C., Ekins S., Williams A. J., Tropsha A. (2018). A bibliometric review
of drug repurposing. Drug Discov Today..

[16] He J., He Y., Qian Y. (2025). Design, synthesis, and biological evaluation
of novel artemisinin-based HDAC inhibitors with antitumor and antimalarial
activities. Bioorg Chem..

[17] Kanyal A., Rawat M., Gurung P., Choubey D., Anamika K., Karmodiya K. (2018). Genome-wide
survey and phylogenetic analysis of histone
acetyltransferases and histone deacetylases of *Plasmodium
falciparum*. FEBS J..

[18] Joshi M. B., Lin D. T., Chiang P. H. (1999). Molecular cloning and
nuclear localization of a histone deacetylase homologue in *Plasmodium falciparum*. Mol.
Biochem. Parasitol..

[19] Engel J. A., Jones A. J., Avery V. M. (2015). Profiling
the anti-protozoal
activity of anti-cancer HDAC inhibitors against Plasmodium and Trypanosoma
parasites. Int. J. Parasitol Drugs Drug Resist..

[20] Sumanadasa S. D. M., Goodman C. D., Lucke A. J. (2012). Antimalarial Activity
of the Anticancer Histone Deacetylase Inhibitor SB939. Antimicrob. Agents Chemother..

[21] Huang Z., Li R., Tang T. (2020). A novel multistage antiplasmodial inhibitor
targeting *Plasmodium falciparum* histone
deacetylase 1. Cell Discovery.

[22] Potluri V., Shandil R. K., Gavara R. (2020). Discovery of FNDR-20123,
a histone deacetylase inhibitor for the treatment of *Plasmodium falciparum* malaria. Malar J..

[23] Darkin-Rattray S. J., Gurnett A. M., Myers R. W. (1996). Apicidin:
A novel antiprotozoal
agent that inhibits parasite histone deacetylase. Proc. Natl. Acad. Sci..

[24] Collins J. E., Lee J. W., Bohmer M. J. (2021). Cyclic Tetrapeptide
HDAC Inhibitors with Improved *Plasmodium falciparum* Selectivity and Killing Profile. ACS Infect
Dis..

[25] Tavares M. T., Krüger A., Yan S. L. R. (2023). 1,3-Diphenylureido hydroxamate
as a promising scaffold for generation of potent antimalarial histone
deacetylase inhibitors. Sci. Rep..

[26] Kanyal A., Deshmukh B., Davies H. (2024). PfHDAC1 is an essential
regulator of *P. falciparum* asexual
proliferation and host cell invasion genes with a dynamic genomic
occupancy responsive to artemisinin stress. mBio..

[27] Waitman K. B., de Almeida L. C., Primi M. C. (2024). HDAC specificity and
kinase off-targeting by purine-benzohydroxamate anti-hematological
tumor agents. Eur. J. Med. Chem..

[28] Waitman K. B., Martin H. J., Carlos JAEG. (2025). Dona Flor and her two
husbands: Discovery of novel HDAC6/AKT2 inhibitors for myeloid cancer
treatment. Comput. Biol. Med..

[29] Porter N. J., Wagner F. F., Christianson D. W. (2018). Entropy
as a Driver of Selectivity
for Inhibitor Binding to Histone Deacetylase 6. Biochemistry.

[30] Wicht K. J., Mok S., Fidock D. A. (2020). Molecular Mechanisms
of Drug Resistance in *Plasmodium falciparum* Malaria. Annu. Rev. Microbiol..

[31] Singh M. K., Tessarin-Almeida G., Dias B. K. M. (2021). A nuclear protein, PfMORC
confers melatonin dependent synchrony of the human malaria parasite *Plasmodium falciparum* in the asexual stage. Sci. Rep..

[32] Chahine, Z. ; Gupta, M. ; Lenz, T. Pf MORC protein regulates chromatin accessibility and transcriptional repression in the human malaria parasite *Plasmodium falciparum* Preprint posted online 2023 10.1101/2023.09.11.557253. PMC11620747 39636094

[33] Andrews K. T., Tran T. N., Fairlie D. P. (2012). Towards histone deacetylase inhibitors
as new antimalarial drugs. Curr. Pharm. Des..

[34] Mohapatra T. K., Nayak R. R., Ganeshpurkar A., Tiwari P., Kumar D. (2024). Opportunities
and Difficulties in the Repurposing of HDAC Inhibitors as Antiparasitic
Agents. Drugs Drug Candidates.

[35] Ekland E. H., Schneider J., Fidock D. A. (2011). Identifying apicoplast-targeting
antimalarials using high-throughput compatible approaches. FASEB J..

[36] Lambros C., Vanderberg J. P. (1979). Synchronization
of *Plasmodium falciparum* Erythrocytic
Stages in Culture. J. Parasitol..

[37] Friesner R. A., Banks J. L., Murphy R. B. (2004). Glide: A New Approach
for Rapid, Accurate Docking and Scoring. 1. Method and Assessment
of Docking Accuracy. J. Med. Chem..

[38] Bowers, K. J. ; Sacerdoti, F. D. ; Salmon, J. K. Molecular Dynamics---Scalable Algorithms for Molecular Dynamics Simulations on Commodity Clusters, Proceedings of the 2006 ACM/IEEE Conference on Supercomputing - SC ’06.; ACM Press, 2006; p 84 10.1145/1188455.1188544.

[39] Lu C., Wu C., Ghoreishi D. (2021). OPLS4: Improving Force Field Accuracy
on Challenging Regimes of Chemical Space. J.
Chem. Theory Comput..

[40] Darden T., York D., Pedersen L. (1993). Particle mesh
Ewald: An *N* ·log­(*N*) method
for Ewald sums in large systems. J. Chem. Phys..

