# Supplementary material for: Repurposing 6‑Anilinopurine Derivatives That Exhibit PfHDAC1 Inhibition and Antimalarial Activity against Asexual and Sexual Stages of Plasmodium falciparum
Source: ACS Omega. 2026 Jul 6;11(28):41385–95. doi: 10.1021/acsomega.5c12744 (PMC13393398; doi:10.1021/acsomega.5c12744)
Supplement: Supplementary file 1 [file ao5c12744_si_001.pdf]

Supporting information

Repurposing 6-Anilinopurine Derivatives That Exhibit PfHDAC1 Inhibition and Antimalarial Activity Against Asexual and Sexual Stages of *Plasmodium falciparum*

Bárbara K. M. Dias[a], Pedro N. Maiolini[a], Natacha Diesca Santos[a], Karoline B. Waitman[b], Maurício T. Tavares[c], João P. F. Verotti[b], Mônica F. Z. J. Toledo[b], Thales Kronenberger[d,e], Roberto Parise-Filho[b*], Célia R. S. Garcia[a*]

[a]Department of Clinical and Toxicological Analyses, School of Pharmaceutical Sciences, University of São Paulo, 05508-000, São Paulo, Brazil

[b]Department of Pharmacy, School of Pharmaceutical Sciences, University of São Paulo, 05508-000, São Paulo, Brazil

[c]Department of Cancer Biology, Dana-Farber Cancer Institute, 02215, Boston, United States.

[d] [1]Interfaculty Institute of Microbiology and Infection Medicine (IMIT), University of Tübingen, Tübingen, Germany. [b]Partner-site Tübingen, German Center for Infection Research (DZIF), 72076, Tübingen, Germany.

[e]School of Pharmacy, Faculty of Health Sciences, University of Eastern Finland, P.O. Box 1627, FI-70211 Kuopio, Finland

[*]**Email**: cgarcia@usp.br

[*]**Email**: roberto.parise@usp.br

Supporting information

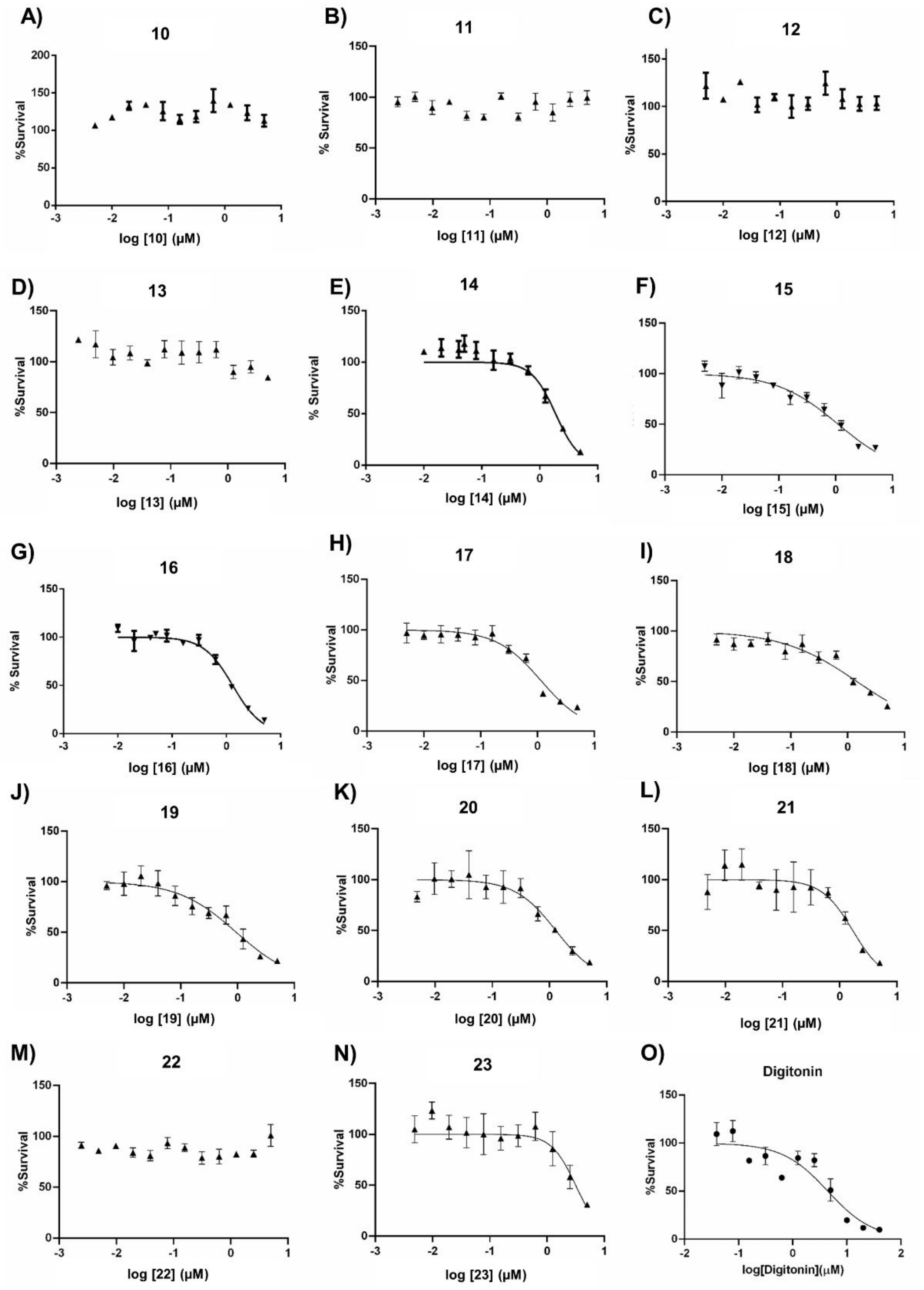

Figure S1. Cytotoxicity effect of compounds in HEK293T cells. Dose-survival curves of HEK293T cells incubated with different concentrations of compounds (A) **10,** (B) **11**, (C) **12**, (D) **13**, (E) **14**, (F) **15,** (G) **16**, (H) **17**, (I) **18**, (J) **19**, (K) **20**, (L) **21**, (M) **22**, (N) **23** for 72 h, ranging from 0.05 to 5 μM. (O) Digitonin was used as a positive control with concentrations ranging from 0.1 to 100 μM. Cytotoxicity was determined by MTT assay. Experiments were performed three independent times in triplicate. Error bars represent the SD.

Supporting information

Table S1. Protein-ligand interaction frequency along the analysed simulation trajectories (5x200 ns).

Moiety	Aas/Ligand	22	12	11	15
Zn^{2+}	E174	100	100	100	100
	H176	68	74	55	45
	E262	100	100	100	100
ZGB	R30	16	12		13
	G136	19	19	20	
	G147		22	36	12
	D262	10			
Linker	H139	25	28	13	27
	H176	24	34	12	30
	L269	10	10	10	10
Cap	E97	19	12		
	C98	14			
	G147	12			
	F148	21		15	11
	F203		20	20	
	R268				13
	Y301		17		
Energy	dG	10.7	-14.7	-12.1	-6.3
	Cou	-37.7	-49.5	-39.2	-40.4
	Hbond	-0.8	-1.1	-1.2	-0.6
	Lipo	-6.2	-13.9	-13.9	-13.9
	LN	2.7	-3.3	-2.7	-1.4

Energy terms are expressed in kcal/mol and ligand efficiency (LN) in kcal/mol.HDAC

Table S2. Effect of 12 and 15 on pf-HDAC1, hHDAC1and hHDAC8 Activity.

Inhibitors	% Inhibition		
	**rh-HDAC1**	**rh-HDAC8**	**pf-HDAC1**
**12**, 1 μM	96	90	96
**12**, 10 μM	99	97	99
**15**, 1 μM	97	73	97
**15**, 10 μM	98	94	98
SAHA, 0.001 μM	10	-	10
SAHA, 0.01 μM	58	-	58
SAHA, 0.1 μM	91	-	91
TSA, 0.01 μM	-	1	-
TSA, 0.1 μM	-	40	-
TSA, 1 μM	-	80	-

HDAC activity assays were performed in duplicates at each concentration. The fluorescent intensity data were analyzed using the computer software, Graphpad Prism. In the absence of the compound, the fluorescent intensity (F_t) in each data set was defined as 100 % activity. In the absence of HDAC1, the fluorescent intensity (F_b) in each data set was defined as 0 % activity. The percent activity in the presence of each compound was calculated according to the following equation: % activity = $(F-F_b)/(F_t-F_b)$, where F =the fluorescent intensity in the presence of the compound.
